# Mechanistic study of celastrol-mediated inhibition of proinflammatory activation of macrophages in IgA nephropathy via down-regulating ECM1

**DOI:** 10.7150/ijbs.99738

**Published:** 2024-10-21

**Authors:** Juanyong Zhao, Haiyang Liu, Qian Chen, Ming Xia, Lili Wan, Weihong Yu, Chenxi Liu, Xiaomiao Hao, Chengyuan Tang, Guochun Chen, Yu Liu, Fang Yuan, Hong Liu

**Affiliations:** Hunan Key Laboratory of Kidney Disease and Blood Purification, Department of Nephrology, The Second Xiangya Hospital, Central South University, Changsha, China.

**Keywords:** IgA Nephropathy, Macrophage, ECM1, Celastrol, Ubiquitination

## Abstract

Increasing evidence suggests that the mononuclear/macrophage system is vital in amplifying the inflammatory cascade in IgA Nephropathy (IgAN). However, the pathogenic mechanism of macrophages in IgAN and targeted treatment strategies still need to be explored. This study found that botanical triterpene celastrol (CLT) effectively alleviated renal lesions, M1-like macrophage infiltration, inflammatory factors production, and improved renal function in IgAN mice. We found that the renal macrophages of IgAN patients had high expression of ECM1, a crucial molecule involved in macrophage inflammatory polarization, positively correlated with the IgAN clinical severity. In murine macrophage Raw 264.7 cells, CLT inhibited macrophage M1-like polarization and the output of TNF-α and IL-6 by downregulating the ECM1/STAT5 pathway. Mechanistically, molecular docking, CESTA, and immunoprecipitation verified that CLT directly bound to ECM1 and increased the ubiquitination of ECM1. Collectively, these results illustrated that CLT inhibited proinflammatory macrophage in IgAN by directly targeting ECM1 to promote ubiquitination degradation of ECM1. Therefore, this study may provide a theoretical basis for exploring the pathogenesis of IgAN and identifying new perspectives for targeted therapy of IgAN.

## Introduction

IgA nephropathy (IgAN) is the most common primary glomerulonephritis in the world. End-stage renal disease (ESRD) will develop in about 30% of IgAN patients 1, 2. The optimal supportive care recommended by the KDIGO guidelines included using renin-angiotensin system inhibitors, lipid-lowering, and lifestyle adjustments. Even with optimal supportive care, many patients nevertheless have considerable proteinuria and are therefore still at risk of developing progressive kidney dysfunction. Additionally, the risks and benefits of immunosuppressive therapy are still under discussion. Recent advancements in IgAN biological agents have produced encouraging results, but the safety and efficacy of these drugs still need to be tested 3, 4. Therefore, effective medications with fewer side effects are urgently needed to treat IgAN. Macrophages are essential for amplifying the inflammatory cascade in IgAN 5, 6. Macrophages were discovered to be the immune cells with the greatest number of differentially expressed genes in the single-cell sequencing investigation conducted in early IgAN mice models 7, but their role is controversial. The primary infiltration type of macrophage in the early stages of renal damage is the pro-inflammatory M1-like phenotype (expression of markers like inducible nitric oxide synthase (iNOS)), which secretes inflammatory mediators and encourages glomerular intrinsic cells and basement membrane destruction. The M2-like phenotype (expression of markers like CD206 and arginase-1 (Arg-1)) at the later phases of repair is important for tissue repair, anti-inflammatory effects, and the prevention of renal fibrosis 8-10. However, there is still a lack of drugs targeting macrophages. Due to the certainly harmful effects of pro-inflammatory macrophages. This study focuses on inhibiting pro-inflammatory macrophages and delaying disease progression in the early stages of IgAN renal injury.

The traditional Chinese medicine *Tripterygium Wilfordii Hook. f.* has been used in treating various kidney diseases, including IgAN, for many years. It effectively reduces hematuria, urinary protein, and renal inflammation 11-13. But their side effects cannot be ignored 14. Celastrol (CLT), a p-quinone methide-containing pentacyclic triterpenoid, is the main effective component of *Tripterygium Wilfordii Hook. F.* Its powerful anti-inflammatory, antiproliferative, and immunosuppressive effects have been demonstrated 15, 16. We have constructed a Tripterygium Ingredient-IgAN targets network and found that CLT is one of the core components of *Tripterygium Wilfordii Hook. F.* for treating IgAN 17. Up to now, reports have shown that CLT can effectively treat glomerular diseases such as mesangial proliferative glomerulonephritis 18, Lupus nephritis 19, diabetes nephropathy 20 by reducing the production of inflammatory cytokines, inhibiting immunity, regulating metabolism and other mechanisms. However, the mechanism of IgAN treatment is still unclear. Resently CLT nanoparticles have been successfully delivered directly to macrophages or kidney intrinsic cells 18, 21, 22 due to the development of nano-drug delivery technology to exert therapeutic effects while limiting toxicity and adverse reactions. Therefore, further exploring the molecular mechanism of CLT in treating IgAN will provide a promising prospect for targeted treatment. Previous studies have shown that CLT can inhibit the M1 polarization of macrophages and induce them to the M2 phenotype, hence reducing inflammation 23, 24.

According to glomerular transcriptomics analysis, Extracellular matrix protein 1 (ECM1) has been reported to be upregulated in IgAN glomerulus 25, 26. It is currently unknown how ECM1 contributes to the progress of IgAN. ECM1 is an important innate immune system regulatory molecule and has been proven to be a crucial protein that supports the development and operation of T cells 27-29 and macrophages 30. In macrophages, increased expression of ECM1 inhibits the phosphorylation of STAT5, making macrophages polarize towards the proinflammatory phenotype and mediating the production of inflammatory cytokines. Interestingly, we discovered that ECM1 had strong binding potential with CLT through molecular docking analysis. ECM1 is a potential target for inhibiting macrophage polarization in IgAN by CLT.

In this study, we speculated that CLT may inhibit M1-like phenotype macrophages by down-regulating ECM1 in IgAN, thereby reducing the production of inflammatory factors and playing a therapeutic role in renal injury.

## Method

### Animals

8-week-old male C57BL/6J mice (weighing 20-25*g*) obtained from Cyagen Biosciences Inc (China, License number: SCXK-(Su)2018-0003) were seeded in a specific pathogen-free condition with standard laboratory diet. After adaptive feeding for a week, the mice were randomly divided into the control group (CON), the IgAN group (IgAN), the control group treated with CLT (CON-CLT), and the IgAN group treated with CLT (IgAN-CLT). To establish the IgAN mouse model, as described in a previous study 17, BSA dissolved in 0.16% hydrochloric acid solution (800 mg/kg) was administered by gavage every two days, CCl4 dissolved in castor oil (1:5) was injected subcutaneously weekly (0.08 ml each) and intraperitoneal biweekly (0.1 ml each). At weeks 6 and 8, LPS (2 mg/kg, Sigma, #L6511) was injected through the tail vein. From week 7, mice received CLT (1 mg/kg/d, Selleck, #NSC 70931) or saline intraperitoneal injection for 21 days. The IgAN model was established at the end of the 10th week. All mice were anesthetized with pentobarbital and euthanasia by cervical dislocation. The kidney tissue, blood, and urine samples were collected. IgA deposition in the glomerulus was visualized through direct immunofluorescence to examine whether the model was established successfully. The animal experiment procedures followed the national guidelines of laboratory animal welfare and they were approved by the Institutional Animal Care and Use Committee at the Second Xiangya Hospital, Central South University (Approval No. 2021509).

### Patients

The enrolled patients were diagnosed with IgAN or Minimal Change disease (MCD) which were confirmed by renal biopsy at the Second Xiangya Hospital of Central South University. The renal biopsy tissues were obtained for subsequent research. The Ethics Committee of the Second Xiangya Hospital of Central South University (Approval No. 2021SNK1123000) approved all procedures.

### Cell culture, Treatment and Transfection

Mouse mononuclear macrophage leukemia Cell line Raw 264.7 was purchased from China Cell Biolabs Company. Cells were cultured in DMEM medium with 10% fetal bovine serum at 37 °C and a 5% carbon dioxide incubator. Cells were treated with the medium containing LPS (1 ug/ml, Sigma, #L6511) and an appropriate concentration of CLT, then harvested at 24 h. LPS was dissolved in PBS, while CLT was dissolved in DMSO. The lentiviral vector pLVX-IRES-Neo-musEcm1-HA (Exp-ECM1), and empty vector were constructed with the company's assistance (Clontech, USA). 293T cells with a confluency of approximately 70-80% were used to prepare for transduction. Lentiviral vectors and Lentivirus packaging plasmid (psPAX2 and pMD2G, Addgen, USA) were transfected into 293T cells by lipofection 2000. After 48-72 h of culture, the vectors were packaged into 293T cells, and the virus supernatant was collected, filtered, and stored at -80 °C. The Raw 264.7 cells were infected by the virus and stably transduced cells screened with puromycin (3 μg/ml). The efficiency of regulated ECM1 expression was measured using real-time PCR and Western blotting.

### Histology and immunohistochemical (IHC) staining

The renal biopsy tissues were soaked in 4% paraformaldehyde, embedded in paraffin, and sliced into 4 μm sections. Periodic acid-Schiff (PAS) staining was performed to observe the renal lesions and infiltration of inflammatory cells, as previously mentioned 31. After antigen retrieval in heated citrate buffer, the slides were exposed to primary antibody F4/80 (1:300, Cell Signaling Technology, #70076T) at 4 °C overnight. Then, the tissues were covered with HRP-linked secondary antibodies (ZSJB-BIO, #PV-9000) for 2 hours and stained with the DAB kit (ZSJB-BIO, #ZLI-9018). The mouse tissue sections were examined by light microscopy (Nikon Tokyo, Japan). Two renal pathologists estimated all renal biopsy specimens. Quantitative analysis was performed with ImageJ software.

### Measurement of urine albumin and creatinine

According to the kit's instructions, urine albumin and urinary creatinine levels were determined using enzyme-linked immunosorbent assay kits (Nanjing Jiancheng Bio, #C035-2-1, #C011-2-1).

### Immunofluorescence

As previously described, the IgA deposition of mouse renal glomerulus was measured by direct immunofluorescence (IF) analysis 32. Using fluorescent primary IgA antibody for detection (1:1000, abcam, #ab307774). For indirect immunofluorescence, Raw 264.7 cells were seeded on the coverslips. After the treatment, cells were fixed with 4% paraformaldehyde before the permeation and blocking. Double indirect IF of the renal biopsies was performed to observe the expression of target molecules. After deparaffinization and rehydration, 4-μm-thick paraffin-embedded sections were in a heated citrate buffer for antigen retrieval. After cooling to room temperature, the sections were blocked with a Blocking Buffer (Abiowell, #AWI0119a) at room temperature for 15 min. Subsequently, incubation with primary antibody CD68 (1:1000, Abcam, #ab303565), iNOS (1:100, Immunoway, #YT3169), CD206 (1:100, Immunoway, #YT5640), ECM1 (1:100, Immunoway, #YT1455), STAT5 (1:100, Immunoway, #YT4452), p-STAT5 (1:100, Immunoway, #YP0613) was performed overnight at 4 °C. Next, sections were incubated with goat anti-mouse IgG conjugated with Alexa Fluor 488 (1:1000, Abcam, #ab150080) and goat anti-rabbit IgG antibody (1:1000, Abcam, #ab150080) conjugated with Alexa Fluor 555 at 37 °C for 2 h. Some experiments use Tyramide Signal Amplification kits (Abiowell, #AWI0692) for fluorescence staining. Anti-fading DAPI was used to visualize the nucleus and seal the samples. We observed CD68, iNOS, CD206, ECM1, STAT5, and p-STAT5 under a fluorescence microscope (Leica, Germany) and performed semi-quantitative analysis by ImageJ software (Media Cybernetics, USA).

### Microarray analysis

The glomerular microdissection sequencing dataset GSE93798 of IgAN patients was downloaded from the GEO database (https://www.ncbi.nlm.nih.gov/gds/). The details of the GSE93798 dataset were as follows: the expression profile platform is GPL22945, which contains 19764 probes and 44 samples, including 22 healthy kidney transplant donor control samples and 20 IgAN samples. The differential expression analysis of microarray data was performed using the limma package in R software (version 3.4.1, Vienna, Austria). The significance of differential mRNA levels was defined by |log2(Fold Change)|>1 and p-value<0.05. The heat maps were created with R software, and the volcano plot was made by the online platform bioinformatics (http://www.bioinformatics.com.cn).

### CCK-8 assay

Cell viability was examined by the CCK8 assay. Raw 264.7 cells were seeded in a 96-well plate at a density of 5000 cells per well. After CLT 0, 0.125, 0.25, 0.5, 0.75, 1.0μmol/L treatment for 24h, cells were incubated with CCK8 solution (TargetMol, #C0005) 10ul per well at 37 °C for 2 h. The absorbance was measured at 450 nm using a microplate reader (Thermo Fisher Scientific).

### Quantitative real-time PCR analysis

Total RNA was extracted from the Raw 264.7 cells or kidney cortexes of mice using Trizol reagent (Accurate Biology, #AG21101). The total RNA was reverse transcribed to cDNA using HiScript III RT Supermix (+gDNA wiper) (Accurate Biology, #AG11728). Quantitative real-time PCR analyses used a LightCycler 96 System (Roche, Germany) with the SYBR Master Mix (Accurate Biology, #AG11759). The quantitative primers of ECM1, Arg-1, iNOS, TNF-α, and IL-6 were designed and synthesized by Sangon Biotech (China) and are listed in Table S1. The results were normalized relative to the expression of β-actin and quantification cycle (Cq) values were calculated using the maximum second derivative method.

### Western blotting (WB)

The cells were ground in RIPA Lysis buffer (Beyotime Biotechnology, China) supplemented with PMSF. The lysate was then centrifuged at 1200 g for 10 min at 4 °C after being broken up three times by ultrasonic to extract the supernatants. The concentration of protein was measured by the BCA protein assay kit (Takara, #T9300A). Then, protein samples were mixed with 5×SDS loading buffer and boiled for 10 min. The samples were separated by SDS-PAGE and transferred to polyvinylidene difluoride (PVDF) membranes. After being blocked with 5% BSA at room temperature for 2 h, the membranes were incubated at 4 °C with the primary antibodies overnight: iNOS (1:2000, Immunoway, #YT3169), IL-6 (1:1000, Abcam, #ab229381), TNF-α (1:2000, Proteintech, #17590-1-AP), Arg-1(1:2000, Proteintech, #16001-1-AP), ECM1 (1:1000, Proteintech, #11521-1-AP; 1:500, Santa Cruz, #sc-365335), STAT5 (1:2000, Immunoway, #YT4452), p-STAT5 (1:2000, Immunoway, #YP0613). After washed with TBST buffer, the membranes were incubated with horseradish peroxidase (HRP)-conjugated secondary antibodies (1:5000, goat anti-rabbit IgG H&L or goat anti-mouse IgG H&L, Proteintech, #SA00001-2, #SA00001-1, #SA00001-19) for 1h at room temperature, the blots of protein were visualized with a chemiluminescence reagent kit and analyzed by ImageJ software.

### Molecular docking

The crystal structure of the ECM1 protein was obtained in the Protein Data Bank (PDB, https://www.rcsb.org/). The 3D structures of CLT were downloaded from PubChem (https://pubchem.ncbi.nlm.nih.gov/). The Autodock Vina 1.1.2 was used to perform molecular docking and calculate the binding affinity. Each calculation generated 10 structures, and the molecular docking output was prioritized according to the frequency of possible ligand-binding sites and free energy score. The docking results of ECM1 proteins and CLT were visualized by PyMOL 2.2.0 software.

### Cellular thermal shift assay

The binding of CLT to potential protein targets was validated with cellular thermal shift assays (CESTA) 33. The total protein of LPS-treated Raw 264.7 cells was extracted, then equal amounts were incubated with CLT (20 μmol/L) or DMSO at 37 °C for 60 min. The mixtures were aliquoted into 6 EP-tubes, each tube containing 80 ul, which were heated to 6 different temperatures of 40 °C, 43 °C, 46 °C, 49 °C, 52 °C, 55 °C for 3 min, then incubated at room temperature and 4 °C for 3 min each. Samples were subjected to Western blotting.

### MG132 treatment protocols

Raw 264.7 cells were pretreated with MG132 for 4 hours at 1 μM, followed by 24 hours of treatment with CLT. At the end of the treatment, cells were harvested and lysed.

### Immunoprecipitation

In brief, Raw 264.7 cells were lysed in a cold IP Lysis buffer containing protease inhibitor cocktail, in addition to 5% cell extracts saved as the input, the rest of the protein lysates were incubated with 1-2 μg anti-ECM1 antibody and IgG (Proteintech, B900620) overnight at 4 °C, and then protein A/G agarose beads (Santa Cruz, #sc-2003) for 6 hours at 4 °C. After incubation, the immunocomplexes were washed six times with Lysis buffer. Bound proteins were eluted by boiling with 2×SDS loading buffer before being analyzed by western blot. The incorporated antibody was ubiquitin (Proteintech, 10201-2-AP, 1:1000).

### Statistical analysis

Data were analyzed and plotted using GraphPad Prism 8.0 and SPSS 23.0 software. All data were expressed as mean ± SD. Two-tailed Student's t-test was used to compare between two groups, and one-way ANOVA and Tukey's multiple comparisons were used to compare between multiple groups. Bivariate correlations were tested using Pearson correlation analysis. All experiments were independently repeated at least three times, and p<0.05 was considered statistically significant.

## Result

### CLT reduces renal lesions and improves renal function in IgAN mice

**Figure 1:**
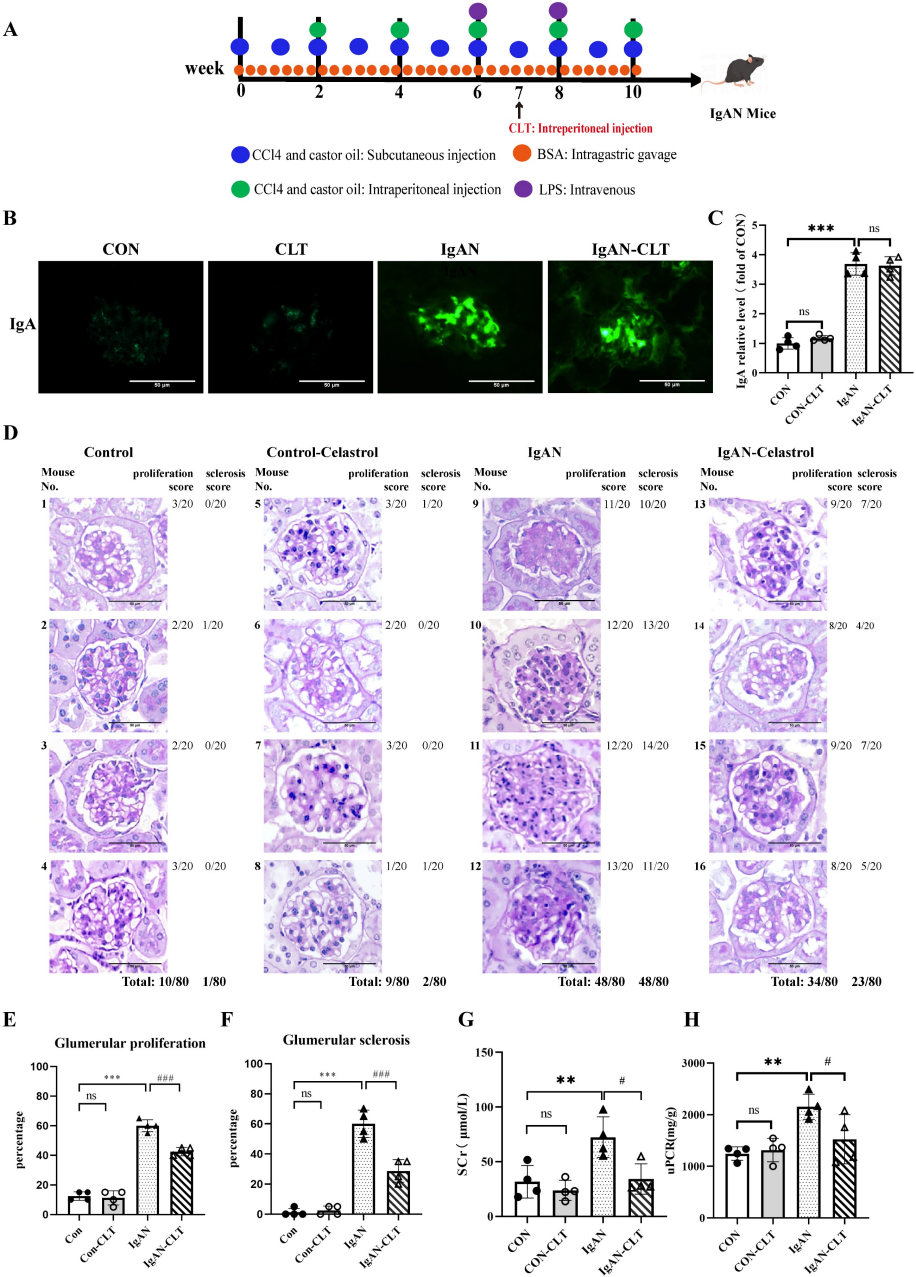
** Celastrol (CLT) reduces renal lesions and improves renal function in IgAN mice.** (A) Schematic diagram of IgAN mice model construction and CLT treatment process. (B) Representative images of glomerular Immunofluorescence staining with IgA (Bars=50μm). (C) The average optical density of IgA was analyzed using ImageJ software. (D) Representative micrographs following periodic acid-Schiff staining (Bars=50μm). Histopathology scoring of (E) glomerular proliferation and (F) sclerosis was based on an evaluation of 80 glomeruli per group (20 glomeruli per animal, 80 glomeruli in total) and is reported as % glomeruli proliferation or sclerosis. The effect of CLT on (G) serum creatinine (SCr) and (H) urinary protein/creatinine ratio (uPCR) in IgAN mice; Statistical differences in multiple groups were determined by one-way ANOVA followed by Tukey's multiple comparisons. All data is represented as mean ± SD, n=4 for each group. Images were captured at ×400 magnification. **: p < 0.01, ***: p < 0.001, vs. CON. #: p < 0.05, ###: p < 0.001, vs. IgAN. ns: p > 0.05.

We established the IgAN mouse model for 10 weeks and started the CLT treatment from week 7. The animal experiment was designed and executed as shown in Figure 1A. Just as previous studies have proven that CLT was an effective leptin sensitizer 34, we also found in our experiments that mice have significantly lost weight since the 7th week of CLT injection (Figure S1). Immunofluorescence staining showed that IgA was significantly deposited in the mesangium of IgAN mice compared with CON. After CLT treatment, the reduction of IgA deposition is not significant (Figure 1B-C), which suggests that CLT may play a role in the pathological process after IgA deposition rather than reducing the production or deposition of pathological IgA. The PAS tissue staining was used to assess glomerular pathology. The tissue sections were quantified by scoring the percent of glomerulus per kidney (20 glomeruli per mouse) exhibiting mesangial cell proliferation, matrix hyperplasia, and global or segmental sclerosis. Histology demonstrated a significantly lower incidence of glomerular mesangial proliferation and sclerosis following 3-week CLT treatments than the IgAN (48/80 vs. 34/80; 48/80 vs. 23/80; Figure 1D-F). To comprehend the renal function of mice, we detected serum creatinine (Scr) and urine protein-creatinine ratio (uPCR) in each of the four groups. As demonstrated in Figure 1G-H, compared to the CON group, the levels of Scr and uPCR increased significantly in the IgAN groups; however, CLT therapy greatly attenuated these increases.

### CLT inhibits M1 polarization of macrophage and release of proinflammatory cytokine in IgAN mice

**Figure 2:**
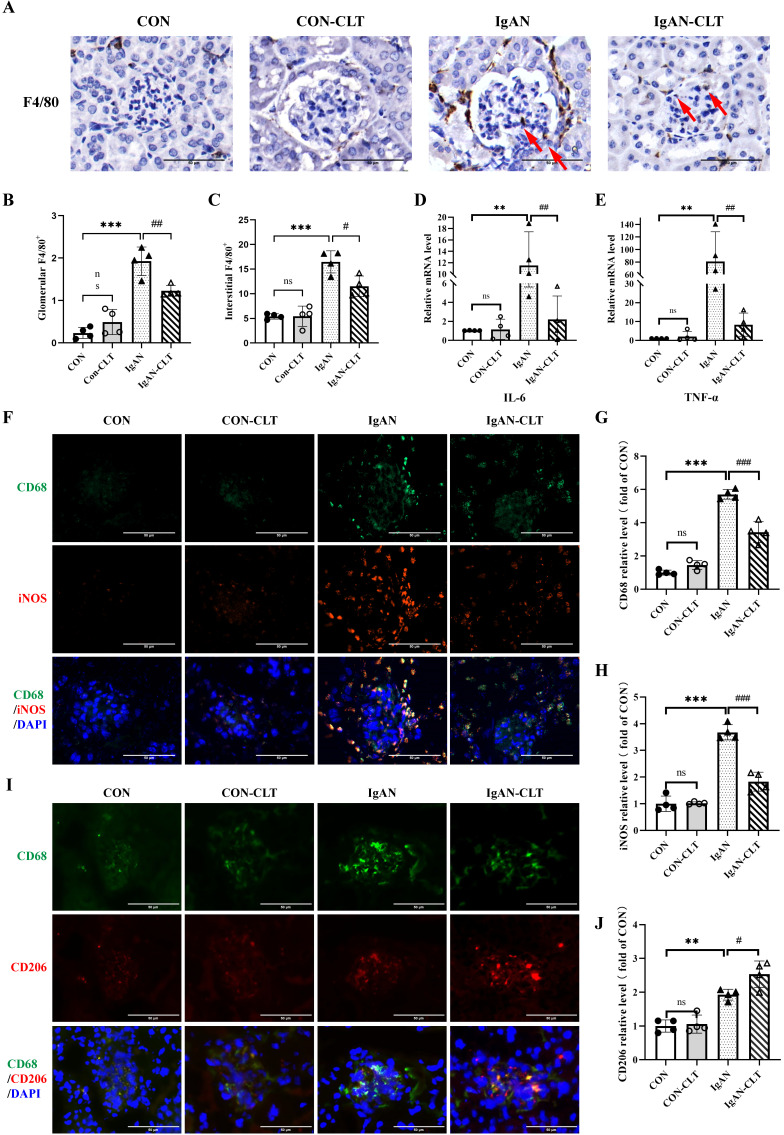
** CLT inhibits M1-like macrophage and the release of proinflammatory cytokine in IgAN mice.** (A) Representative images of F4/80 immunohistochemical in each group kidneys (Bars=50μm), with red arrows indicating F4/80^+^macrophages in the glomerulus. (B, C) The quantity of F4/80^+^ cells in the glomerulus and renal interstitium. (D) IL-6 and (E) TNF-α mRNA levels in the mouse renal cortex. (F) Representative images of double immunofluorescence of CD68 and iNOS in each group of mice kidneys (Bars=50μm). The relative average fluorescence intensity of (G) CD68 and (H) iNOS was analyzed by ImageJ software. (I) Representative images of double immunofluorescence of CD68 and CD206 in each group of mice kidneys (Bars=50μm). (J) The relative average fluorescence intensity of CD206 was analyzed by ImageJ software. Statistical differences in multiple groups were determined by one-way ANOVA followed by Tukey's multiple comparisons. All data were presented as mean ± SD. n=4 for each group. Images were captured at ×400 magnification. **: p < 0.01, ***: p < 0.001, vs. CON. #: p < 0.05, ##: p < 0.01, ###: p < 0.001, vs. IgAN. ns: p > 0.05.

To evaluate CLT alleviates renal inflammation and macrophage infiltration in IgAN mice, the distribution of inflammatory cells in renal was determined by immunohistochemistry (IHC). Compared with the CON mice, there was significant infiltration of F4/80^+^ macrophages in IgAN mice renal (Figure 2A-C). Most macrophages are distributed in the renal interstitium and congregated outside Bowman's bursa. IgAN-CLT mice exhibited significantly reduced infiltration of these macrophages in renal tissues compared to the IgAN mice. The results of real-time PCR (Figure 2D-E) show that mRNA levels of proinflammatory cytokines IL-6 and TNF-α in IgAN mice renal tissues were sensibly increased. After being treated by CLT, expression of them was inhibited. CLT has been shown to inhibit macrophage proinflammatory polarization 21. To determine the effects of CLT on macrophage polarization in IgAN, the expression of the CD68, iNOS, and CD206 was subsequently examined by double immunofluorescence staining in mice kidney tissue. The proportion of M1-like macrophages was significantly higher in the IgAN group, compared with the CON group and the number of macrophages decreased considerably after CLT treatment (Figure 2F-H). Interestingly, CLT somehow enhanced the expression of CD206 in macrophages (Figure 2I-J).

### CLT suppressed proinflammatory macrophage polarization in Raw 264.7 cells

**Figure 3:**
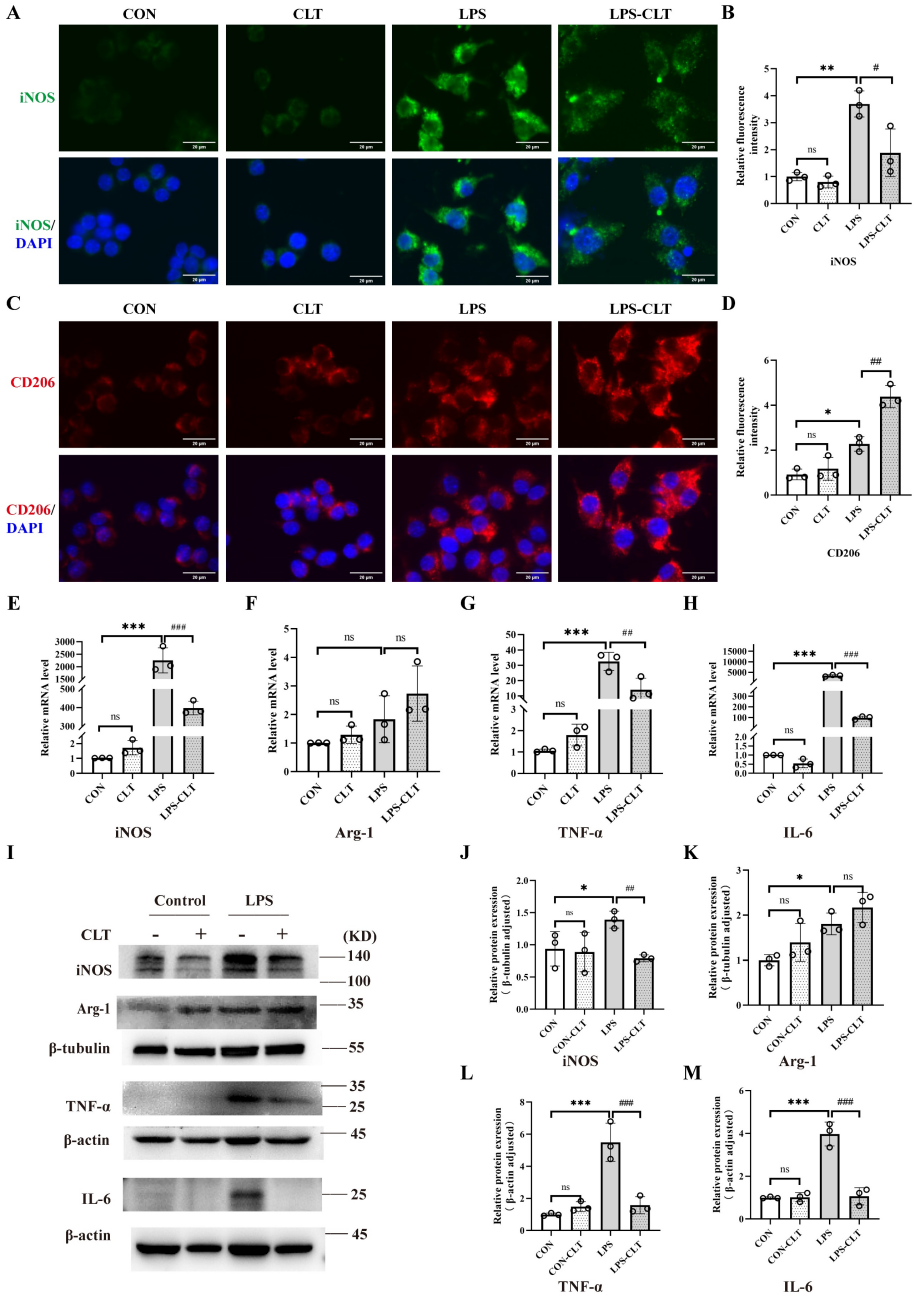
** CLT suppressed proinflammatory macrophage polarization in Raw 264.7 cells.** (A-B) Representative images (Bars=10μm) and quantitative analysis of iNOS immunofluorescence in Raw 264.7. (C-D) Representative images (Bars=10μm) and quantitative analysis of CD206 immunofluorescence in Raw 264.7. (E-H) The levels of mRNA and (I-M) protein of iNOS, Arg-1, TNF-α, and IL-6 in LPS-induced Raw 264.7 cells treated with CLT. Statistical differences in multiple groups were determined by one-way ANOVA followed by Tukey's multiple comparisons. All data were presented as mean ± SD. n=3 for each group. *: p < 0.05, **: p <0.01, ***: p < 0.001, vs. CON. ##: p < 0.01, ###: p < 0.001, vs. LPS. ns: p > 0.05.

To clarify the impact of CLT on macrophages, the macrophage M1 and M2 polarization indicators in the mouse macrophage cell line Raw 264.7 were measured. The CLT concentration gradient was set to 0 μmol/L, 0.125 μmol/L, 0.25 μmol/L, 0.5 μmol/L, 0.75 μmol/L, and 1.0 μmol/L to ascertain the safe concentration of CLT therapy Raw 264.7 cells. After 24 hours of intervention with CLT, cell viability was detected by CCK8 assay. Raw 264.7 cells were inhibited growth significantly with CLT concentration rise beyond 0.75 μmol/L (Figure S2A). Consistently, the Raw 264.7 cells intervened by LPS (1μg/ml), and the iNOS protein expression level was detected at the concentrations of 0, 0.125, 0.25, and 0.5μmol/L CLT, as shown in Figure S2B-C. When the CLT concentration was 0.5μmol/L, the level of iNOS protein was at its lowest. According to these findings, 0.5μmol/L of CLT is the safest and most efficient concentration to suppress LPS-induced Raw 264.7. Cellular immunofluorescence staining showed CLT inhibited expression of iNOS in LPS-induced macrophage (Figure 3A-B) and enhanced expression of CD206 (Figure 3C-D). Real-time PCR (Figure 3E-H) and WB (Figure 3I-M) examination showed that high expression of pro-inflammatory cytokines, including iNOS, TNF-α, and IL-6 in LPS-induced Raw 264.7 cells were reduced effectively by CLT, while upregulated the level of M2 polarization biomarker Arg-1 mRNA and protein. In addition, light microscopy of LPS-induced Raw 264.7 cells stimulated with CLT is shown in Figure S3: LPS stimulation increases cell volume and produces pseudopodia. After CLT intervention, the morphological changes of cells are reversed. These results indicated that CLT inhibited LPS-induced macrophage polarization toward the M1 phenotype and slightly enhanced polarization toward the M2 phenotype.

### ECM1 is highly expressed in IgAN macrophages and positively correlated with IgAN clinical severity

**Figure 4:**
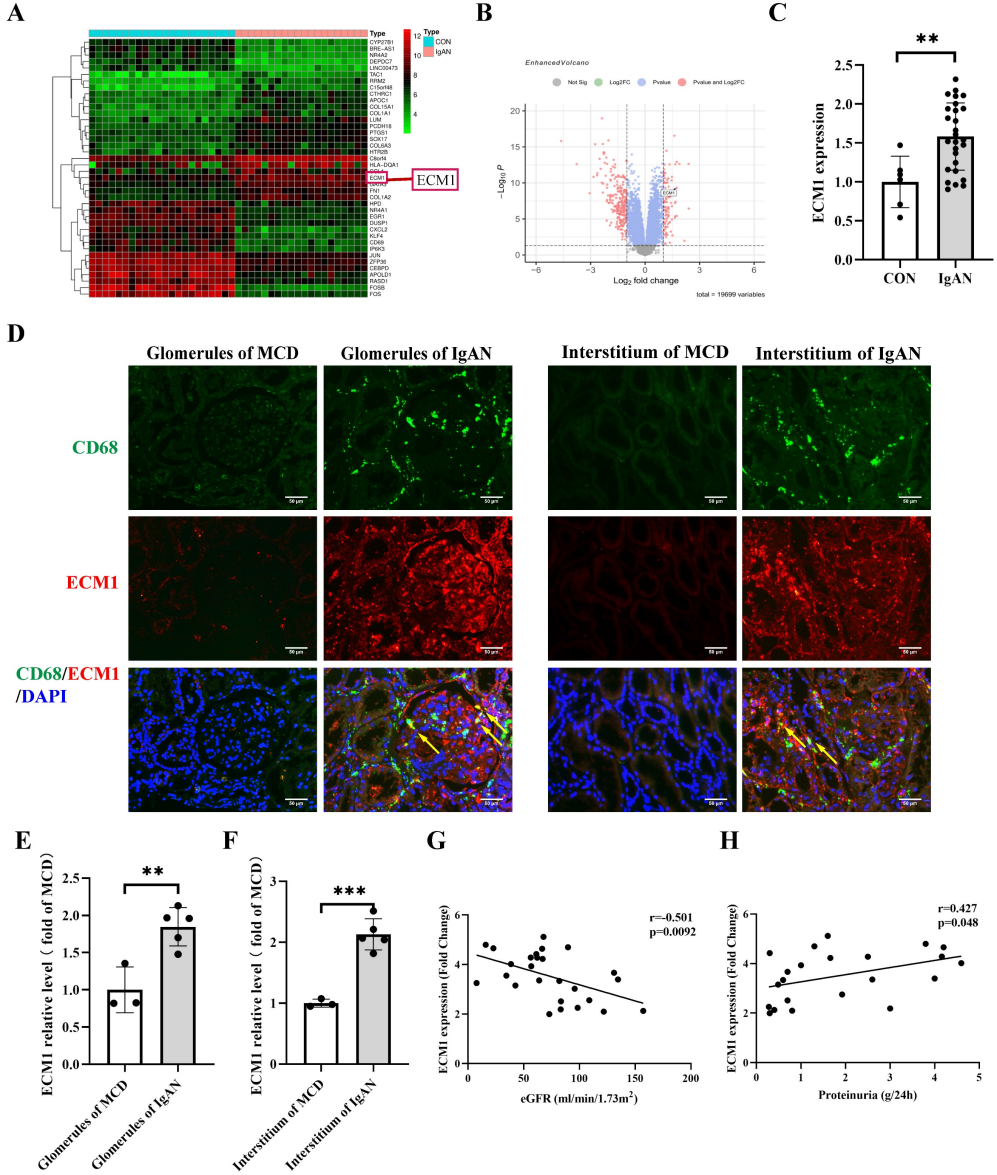
** ECM1 is highly expressed in IgAN macrophages and positively correlated with IgAN clinical severity.** (A) Heat map of top 20 differential genes identified by integrated analysis of the GEO datasets. Red areas represent highly expressed genes and green areas represent lowly expressed genes in glomeruli from IgAN subjects compared with healthy living donors. (B) Volcano plot analysis identifies DEGs. The right red dots represent upregulated genes, and the left red dots represent downregulated genes in glomeruli from IgAN subjects compared to healthy living donors. (C) The levels of ECM1 mRNA in IgAN patients compared to control (p < 0.01, Fold Change = 2.439). (D) Representative images of double immunofluorescence of ECM1 and CD68 in IgAN patients glomerulus and tubulointerstitium (Bar=50μm). (E-F) The relative average fluorescence intensity of ECM1 was analyzed using ImageJ software. (G) Correlation analysis between eGFR and ECM1 transcription levels in IgAN patients (n=26), and (H) correlation analysis between 24-hour urine protein and ECM1 transcription levels (n=22). Statistical differences between the two groups were analyzed using the student's t-test analysis. All data were presented as mean ± SD. Images were captured at ×400 magnification. **: p < 0.01, ***: p < 0.001, vs. CON or MCD.

The dataset GSE93798 from the GEO database was analyzed to obtain differentially expressed genes in human kidney biopsy tissues (22 control vs 20 IgAN). ECM1 is one of the top 20 up-regulated genes (Figure 4A-B). The glomerular sequencing data from the Nephroseq database shows that IgAN patients had considerably higher levels of ECM1 mRNA (Figure 4C). Notably, the important role of ECM1 in preserving M1 polarization in macrophages has been reported 30. Therefore, we focused on its role in macrophages in IgAN renal tissue. We verified the above results using renal biopsy specimens from our hospital. Double immunofluorescence staining of ECM1 and CD68 in patients with IgAN showed that ECM1 was mainly expressed in the mesangium and macrophage, partially overlapping with CD68 in the glomerulus and renal interstitium (Figure 4D-F). Glomerular sequencing data and clinical data of IgAN patients (n=27) were mined from the Nephroseq database. Pearson's bivariate correlation analysis showed that eGFR was negatively correlated with the transcription level of ECM1 (r=-0.501, p=0.0092) (Figure 4G). 24h urinary protein levels were positively correlated with ECM1 transcription levels (r=0.427, p=0.048) (Figure 4H). It is suggested that the abnormal expression of ECM1 may be involved in the progression of IgAN.

### CLT regulated macrophage M1-like polarization and release of proinflammatory cytokine by ECM1

**Figure 5:**
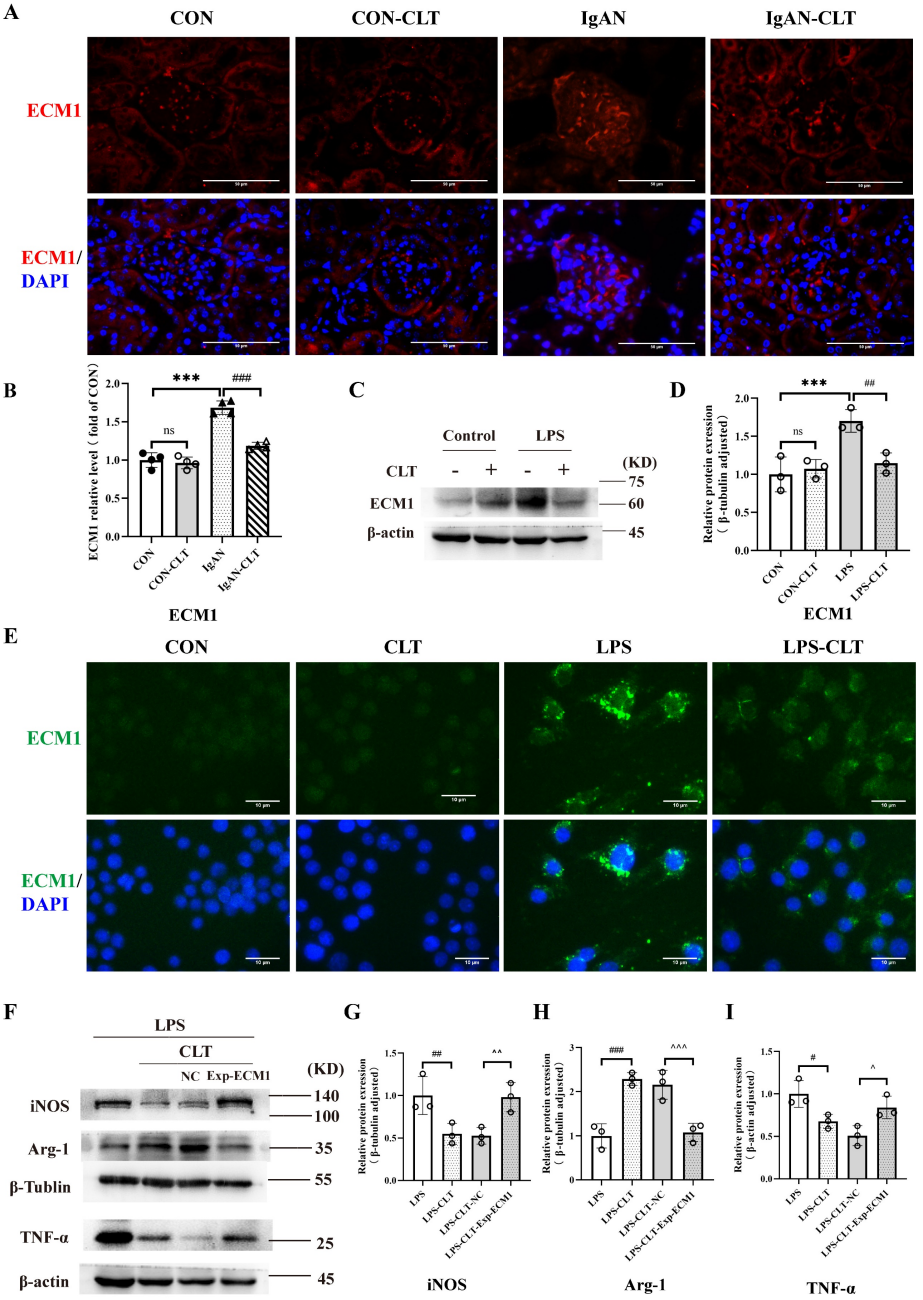
** CLT regulated macrophage M1-like polarization and release of proinflammatory cytokine by ECM1.** (A) Representative images of ECM1 immunofluorescence staining in each group of mice kidneys (Bars=50μm). (B) The relative average fluorescence intensity of ECM1 was analyzed using ImageJ software. (C-D) Immunoblot analyses and quantitative determination of ECM1 in LPS-induced Raw 264.7 cells treated with CLT. (E) Immunofluorescence of ECM1 in Raw 264.7 cells (Bars = 10 μm). (F-I) Representative western blots and quantitative analysis of iNOS, Arg-1, and TNF-α in ECM1-overexpression Raw 264.7 cells. Statistical differences in multiple groups were determined by one-way ANOVA followed by Tukey's multiple comparisons. All data were presented as mean ± SD, n=4 for each group of mice, n=3 for each group of cells. Images were captured at ×400 magnification. *: p<0.05, ***: p < 0.001, vs. CON. #: p < 0.05, ##: p < 0.01, vs. LPS. ^p < 0.05, ^^p < 0.01 vs. LPS-CLT-NC. ns: p > 0.05.

Consistent with the renal pathological results of the patient, the fluorescence intensity of ECM1 in the glomerulus of IgAN mice was significantly increased compared with the CON group. The fluorescence intensity of ECM1 in IgAN-CLT mice was decreased compared with that in the IgAN group, suggesting that CLT could inhibit the expression of ECM1 protein in the kidneys of IgAN mice (Figure 5A-B). WB (Figure 5C-D) and IF (Figure 5E) showed that the ECM1 protein level increased in LPS-induced Raw 264.7 cells while CLT reduced its expression. As the extracellular matrix protein, LPS-induced macrophage secretion of large amounts of ECM1 into the extracellular space was observed. In addition, we constructed ECM1 overexpressing cell lines using lentiviruses. The efficiency of ECM1 overexpression was measured using real-time PCR and WB (Figure S4). Arg-1 raised by CLT was again reduced, and iNOS and TNF-α decreased by CLT were again raised in the ECM1 overexpressed cells (Figure 5F-I).

### CLT inhibited macrophage M1-like polarization via the ECM1/STAT5 pathway

**Figure 6:**
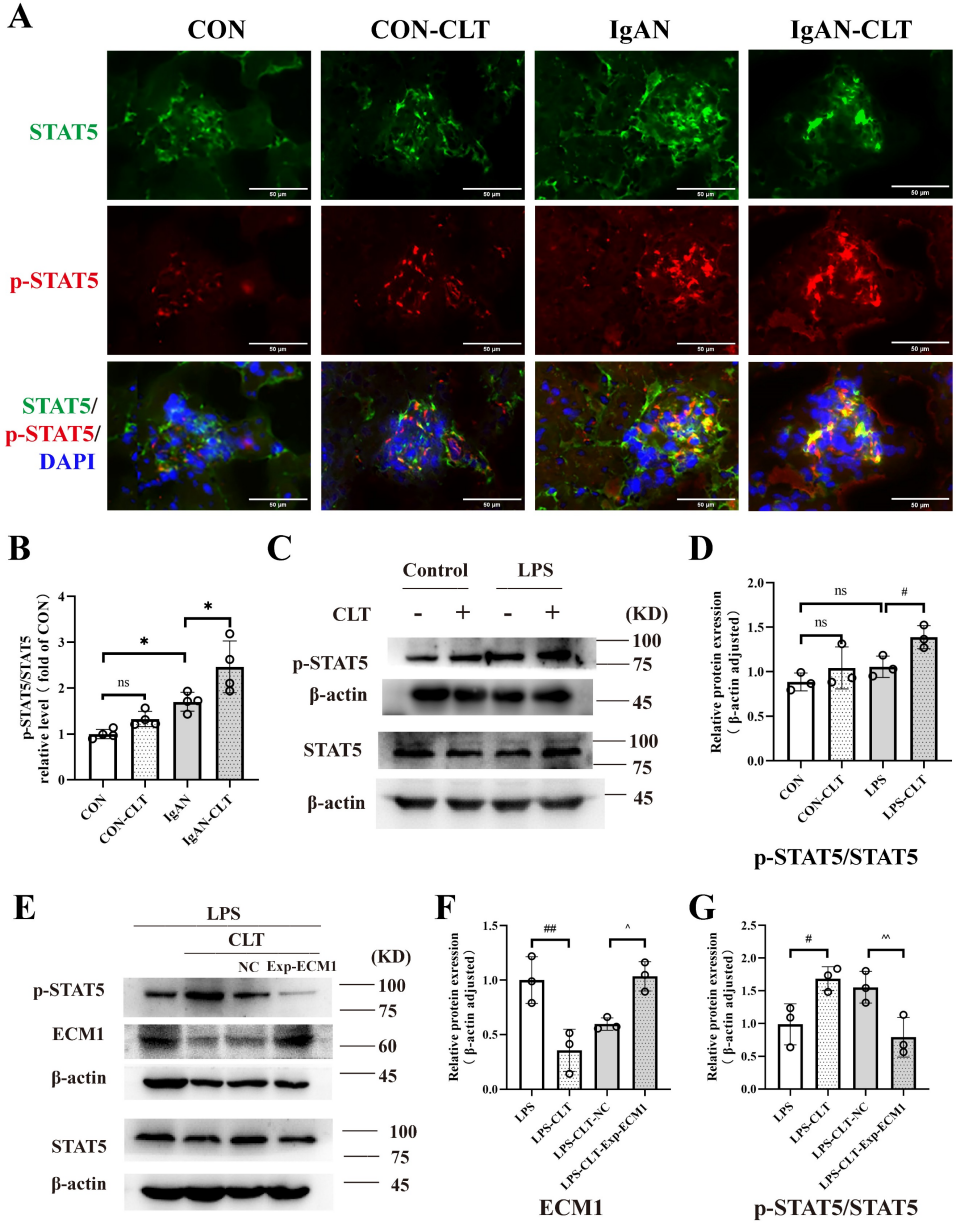
** CLT inhibited macrophage M1-like polarization via the ECM1/STAT5 pathway.** (A) Representative images of STAT5 and p-STAT5 immunofluorescence staining in each group of mice kidneys (Bars=50μm). (B) The relative average fluorescence intensity of p-STAT5/STAT5 was analyzed by ImageJ software.(C) Representative western blots and (D) quantitative analysis of STAT5 and p-STAT5 in LPS-induced Raw 264.7 cells treated with CLT. (E) Representative western blots and (F-G) quantitative analysis of ECM1, STAT5, and p-STAT5 in ECM1-overexpression Raw 264.7 cells. Statistical differences in multiple groups were determined by one-way ANOVA followed by Tukey's multiple comparisons. All data were presented as mean ± SD, n=4 for each group of mice, n=3 for each group of cells. #: p < 0.05, ##: p < 0.01, vs. LPS. ^p < 0.05, ^^p < 0.01 vs. LPS-CLT-NC. ns: p > 0.05.

ECM1 is highly expressed in tissues infiltrated by macrophages and inflammatory cells. The downregulation of ECM1 in macrophages leads to the phosphorylation of STAT5, thereby upregulating the expression of Granulocyte-macrophage colony-stimulating factor (GM-CSF) / Arg-1 and preventing polarization of macrophage M1 phenotype 30. Therefore, we investigated whether CLT upregulates the phosphorylation of STAT5 to inhibit the proinflammatory macrophage. Immunofluorescence staining revealed that p-STAT5 and STAT5 were abundantly expressed within the IgAN mice glomerulus, and p-STAT5 expression increased after CLT treatment (Figure 6A-B). As shown in Figure 6C-D, the p-STAT5 / STAT5 ratios were significantly increased in CLT treatment LPS-induced macrophage. Meanwhile, ECM1 overexpression downregulated p-STAT5/STAT5 in LPS-CLT Raw 264.7 cells (Figure 6E-G). These results indicated that CLT inhibits the proinflammatory macrophages via ECM1/STAT5.

### CLT induces ubiquitin-mediated ECM1 degradation

**Figure 7:**
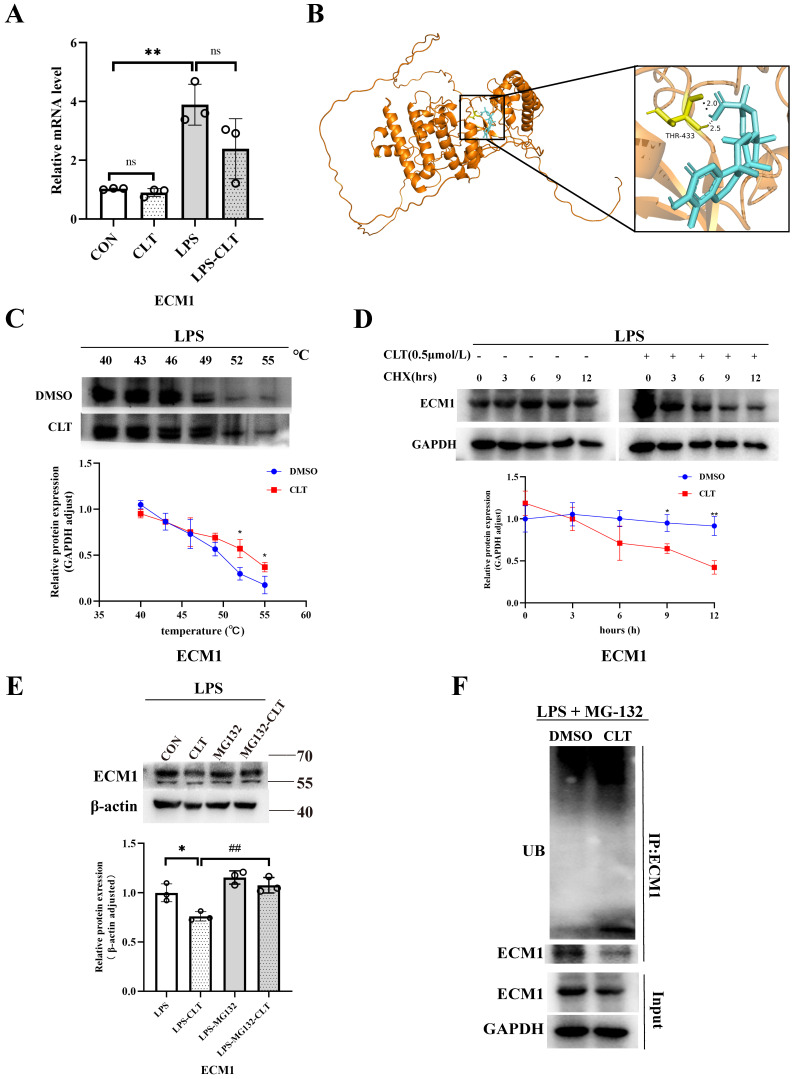
** CLT induces ubiquitin-mediated ECM1 degradation.** (A) The mRNA level of ECM1 in LPS-induced Raw 264.7 cells treated with CLT. (B) 3D Docking conformations of ECM1 and CLT. (C) Raw 264.7 cells under LPS exposure were incubated with CLT or DMSO, and cellular thermal shift assays (CETSA) analyzed the thermal stabilization of ECM1 protein at different temperatures. The line graph presentation of quantitative analysis of ECM1 protein expression. (D) Raw 264.7 cells were treated with CLT under LPS exposure, treated with CHX (200 ng/mL), and collected at the indicated times for western blotting. The line graph presentation of quantitative analysis of ECM1 protein expression. (E) Representative western blots and quantitative analysis of ECM1. Raw 264.7 cells under LPS exposure were pretreated with MG132, followed by treatment with CLT. (F) Raw 264.7 cells under LPS exposure were treated with CLT and MG132. ECM1 was immunoprecipitated with an anti-ECM1 antibody, and the immunoprecipitates were probed with an anti-ubiquitin (UB) antibody. Statistical differences between two groups were analyzed by student's t-test analysis, and differences in multiple groups were determined by one-way ANOVA followed by Tukey's multiple comparisons. All data were presented as mean ± SD, n=3 for each group. *: p<0.05, **: p < 0.01, vs. CON OR LPS. ##: p < 0.01, vs. CLT OR LPS-CLT.

Moving forward, we aim to examine the molecular mechanism of CLT-mediated downregulation of ECM1. ECM1 mRNA level decreased during CLT treatment, but this change was not statistically significant (Figure 7A), indicating that CLT may do so by altering protein levels rather than blocking ECM1 expression at the transcriptional level. Molecular docking analysis showed the possibility that CLT binds to THR-433 of ECM1. The predicted binding energy is -7.51 kcal/mol (Figure 7B). As further confirmation of the combination, The CETSA experiment demonstrated that CLT increased the thermal stability of ECM1 compared to the control solvent (Figure 7C). Cycloheximide (CHX) was applied to Raw 264.7 cells to prevent protein synthesis, and protein levels of ECM1 at different intervention times were detected. The half-life of the ECM1 was drastically lowered by CLT treatment (Figure 7D), suggesting that CLT reduces the ECM1 proteins' stability. Recently, CLT has been proven to be an E3 ubiquitin ligase recruiter 35.

In addition, the reduction in ECM1 protein levels by CLT was blocked by the proteasome inhibitor MG132 in LPS-induced Raw264.7 cells (Figure 7E), indicating that CLT might cause the degradation of ECM1 protein via the ubiquitin-proteasome system (UPS). To verify that CLT induces degradation of the ECM1 protein through the UPS pathway, the levels of polyubiquitylated ECM1 were assessed in ECM1 overexpressed cells. As shown in Figure 7F, ECM1 polyubiquitylation increases with CLT therapy. When combined, these findings suggested that CLT treatment induces ECM1 ubiquitin-mediated degradation.

## Discussion

Macrophages are involved in the IgAN inflammatory response as executors and catalysts. Numerous investigations have demonstrated that macrophages are connected to the advancement of IgAN. CD68^+^ macrophages are positively associated with serum creatinine of IgAN, proteinuria, and worse disease outcomes of IgAN 36, 37. Depletion of macrophages alleviates proteinuria and renal function in immune complex-mediated glomerulonephritis by decreasing the expression of inflammatory cytokines and adhesion molecules (e.g., iNOS, VCAM-1) 38. The pathological manifestations of IgAN are also associated with distinct sites of macrophage infiltration. Interstitial macrophage infiltration is associated with tubular atrophy, interstitial fibrosis, and the severity of proteinuria, whereas glomerular macrophage infiltration is associated with crescent formation 39. The infiltrating macrophage phenotypes vary according to the stage of renal damage. The iNOS^+^ M1-like macrophages infiltrated the kidney in the first 48 hours after acute kidney injury, while Arg-1^+^ M2-like macrophages dominated in the later time. The M1-like macrophages polarized to the M2-like when the injured kidney started to repair 10. The former produce proinflammatory cytokines that are generally considered harmful and contribute to the progression of kidney damage. Therefore, this study focused on the therapeutic mechanism of inhibiting proinflammatory macrophages in the early progression of IgAN renal inflammatory injury.

To simulate the pathological situation at the peak of the progression of IgAN glomerular inflammation as far as possible, this study appropriately shortened the modeling time from 12 weeks to 10 weeks based on the previous experience of our research group in establishing IgAN mouse models 40, so that the execution time node was close to the time of LPS injection into the tail vein (week 8). We observed that M1-like macrophages were significantly increased in IgAN mice renal interstitium at this stage of disease, especially surrounding Bowman's capsule. At the same time, the infiltration of macrophages in the glomerulus also increased, but the amount is quite minimal. Previous studies have found that there are more CD68^+^ macrophages infiltrating glomerulonephritis with Baumann's capsule breaks than there are in the glomerulus without such breaches. Our results may be attributed to the preservation of the complete Bowman's capsule 41. However our method of building mouse models made it difficult to cause Bowman's capsule rupture.

CLT is one of the most bioactive and widely studied active components of *Tripterygium Wilfordii Hook. f.*. Cell Journal listed CLT as the most promising traditional Chinese medicine components for developing into modern medicine 42. CLT has been proven effective for mesangial proliferative glomerulonephritis 16, obesity-related nephropathy 43, and vascular calcification in Chronic Kidney Disease (CKD) 44 by inhibiting inflammatory cell infiltration, reducing cytokine production and inhibiting oxidative stress. CLT has also been reported to alleviate murine lupus nephritis via inducting CD4^+^Foxp3^+^ regulatory T cells. 19. In addition, CLT also has a significant effect on renal fibrosis 44. However, the mechanism of CLT treatment for IgAN is still unclear. This study found that CLT effectively alleviated renal lesions and macrophage infiltration and improved renal function in IgAN mice after 3-weeks of intraperitoneal injection. In addition, after CLT intervention, the expression of iNOS, IL-6, and TNF- α was decreased, and the expression of CD206 and Arg-1 was increased *in vivo* and LPS-induced Raw 264.7 cells. Combined with these results, it is speculated that CLT plays a therapeutic effect on IgAN by inhibiting the polarization of M1-type macrophages and promoting the polarization of M2, reducing the production of inflammatory mediators.

To further explore the mechanism of CLT treatment of IgAN, GEO analysis was applied to screen the key gene ECM1 in IgAN. We found ECM1 was highly expressed in both glomerular and tubulointerstitium macrophages of IgAN patients, and the increased expression of ECM1 in IgAN was positively correlated with clinical severity via correlation analysis. ECM1 is a highly expressed gene reported in glomerular sequencing studies of IgAN patients 26 and single-cell sequencing studies of IgAN mice 25, nevertheless, no studies on the mechanism of action of ECM1 in IgAN have been published since then. Moreover, molecular docking technology predicted that ECM1 was a potential target of CLT. ECM1 is mainly secreted into the extracellular matrix and is also present in the nucleus and cytoplasm. The kidney is a primary parenchymal organ with the highest expression of ECM1 45. ECM1 has recently been recognized as an important regulatory molecule of the immune system. Helper T (Th) cells and macrophages were the immune cells that expressed the highest 30. ECM1 is a key molecule in Th cell maturation, which can bind to IL-2 receptors to inhibit the IL-2 signaling pathway and affect Th cell migration 28. It can also be promoted by down-regulating the phosphorylation level of STAT5 to regulate the production of antibodies by germinal center B cells 27. In macrophages, GM-CSF is inhibited by ECM1 activation, which can prevent STAT5 from being phosphorylated. Thereby this impairs the transcriptional activity of STAT5, reduces the expression of Arg-1, causes macrophage polarization towards the M1 phenotype, and mediates the production of inflammatory cytokines 30. Our experimental results showed that the expression of ECM1 in IgAN mice decreased significantly after CLT treatment. *In vitro* experiments further confirmed that the ECM1/STAT5 pathway was activated in Raw 264.7 cells induced by LPS, and CLT intervened to inhibit the expression of ECM1 and increase the phosphorylation of STAT5. Subsequently, it was found that overexpression of ECM1 promoted M1 polarization of macrophages and weakened the anti-inflammatory effect of CLT.

CLT has been proven to be a broad cysteine-targeting E3 ubiquitin ligase warhead for recruiting E3 ubiquitin ligases that lead to the degradation of target proteins 35, 46. Recent studies indicated that CLT binds to Nur77 and promotes its interaction with the E3 Ubiquitin Ligase TRAF2, which induces Nur77 ubiquitination 47. In the current study, we proved that CLT directly binds to ECM1 protein, inducing ECM1 ubiquitination and leading to proteasome degradation.

There are some limitations in this study. The CLT treatment dose for IgAN mice in this study was 1 mg/kg/day, based on CLT treatments for other chronic kidney diseases 43. A gradient concentration of CLT should be applied for mouse treatment. This study could include multiple time points for examining blood and urine samples from mice. The limited modeling process and flawed techniques also resulted in a relatively small number of mice. Furthermore, the sample size of IgAN patients was limited, with a dearth of patients suffering from other types of kidney diseases. We will enroll more patients with IgAN and other kidney diseases and refine our animal experimental methods in the future. And we are developing ECM1 gene-edited mice to further investigate the mechanism by which CLT targets ECM1 and the role of ECM1 in IgAN.

In conclusion, our study investigated the therapeutic effects of CLT to alleviate IgAN renal inflammatory damage and inhibit M1-like macrophages. CLT downregulates ECM1 to increase STAT5 phosphorylation, thereby inhibiting the expression of inflammatory factors. The inhibitory effect of CLT might result from its targeting of ECM1, which increases ECM1's ubiquitination and destruction. The findings provide new theoretical future targeted therapy of IgAN.

## Supplementary Material

Supplementary figures and table.

## Figures and Tables

**Figure 8:**
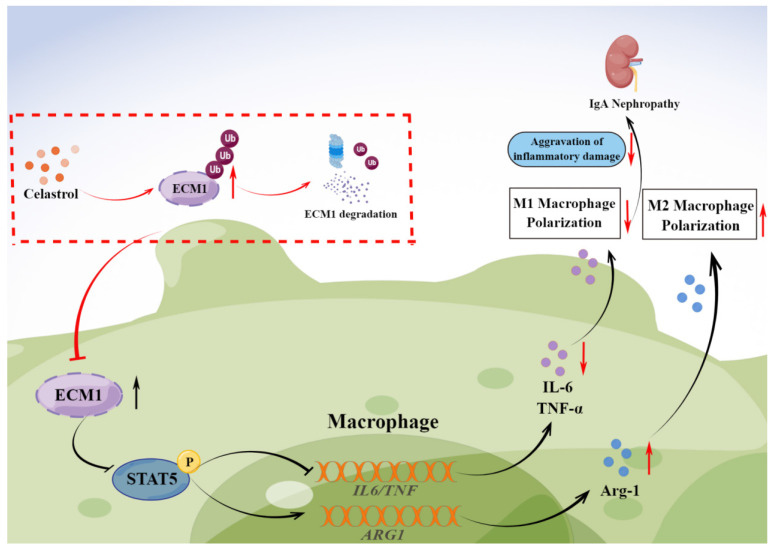
** Graphical abstract.** CLT promotes the ubiquitination and degradation of ECM1 in IgAN macrophages to regulate STAT5 phosphorylation, leading to decreased secretion of IL-6 and TNF-α and upregulation of Arg-1 expression, thereby promoting the transformation of pro-inflammatory M1-like macrophages to reparative M2-like macrophages and reducing inflammatory damage in IgAN.

## Abbreviations

IgAN: IgA Nephropathy
CLT: celastrol
ESRD: End stage renal disease
ECM1: Extracellular matrix protein 1
MCD: Minimal Change disease
iNOS: inducible nitric oxide synthase
Arg-1: arginase-1
GM-CSF: Granulocyte-macrophage colony-stimulating factor
CHX: Cycloheximide
UPS: ubiquitin-proteasome system
CKD: Chronic Kidney Disease
