# Supplementary material for: Mechanistic study of celastrol-mediated inhibition of proinflammatory activation of macrophages in IgA nephropathy via down-regulating ECM1
Source: Int J Biol Sci. 2024 Oct 21;20(14):5731–46. doi: 10.7150/ijbs.99738 (PMC11528456; doi:10.7150/ijbs.99738)
Supplement: Supplementary file 1 — Supplementary figures and table. [file ijbsv20p5731s1.pdf]

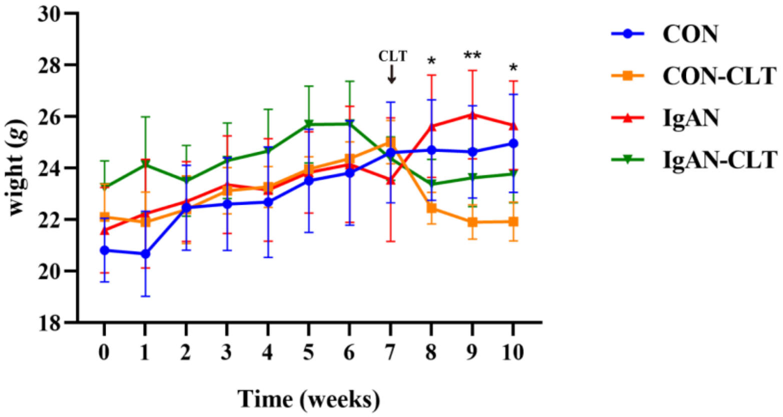

Figure S1 Line chart of mice body weight change. Statistical differences in multiple groups were determined by one-way ANOVA followed by Tukey's multiple comparisons. All data is represented as mean ± SD, n=4.

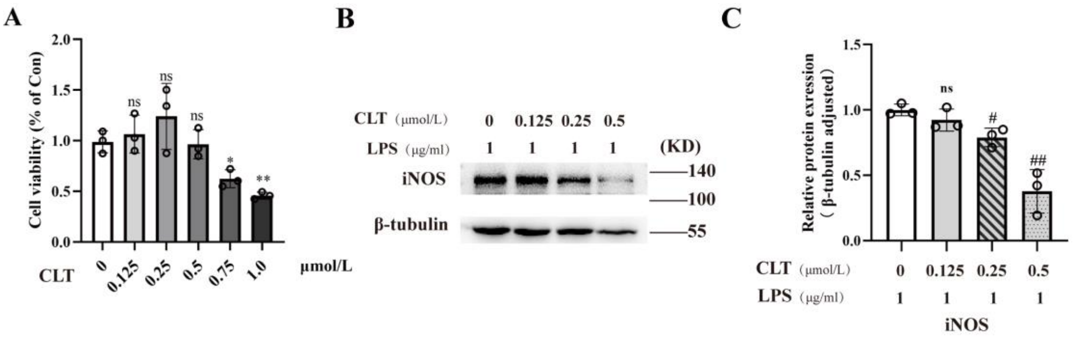

Figure S2 The optimal concentration of CLT in Raw 264.7 cells. (A) Detected cell viability to screen appropriate intervention concentrations by the CCK8 method. (B) Immunoblot analyses and (C) quantitative determination of iNOS in the LPS-induced Raw 264.7, set the CLT concentration gradient to 0, 0.125, 0.25, and 0.5 μmol/L. Statistical differences between treatment groups and CON group were analyzed by student's t-test analysis. All data is represented as mean ± SD, n=3.***: $p < 0.001$, vs. CON. #: $p < 0.05$, ##: $p < 0.01$, vs. LPS. ns: $p > 0.05$.

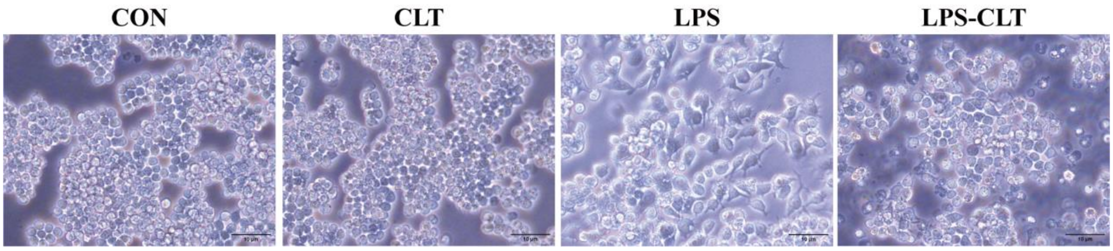

Figure S3 Morphological changes in Raw 264.7 cells. Treatment of LPS-induced Raw 264.7 cells without or with CLT for 24 h. Representative light microscopy images are presented. Images were captured at ×200 magnification.

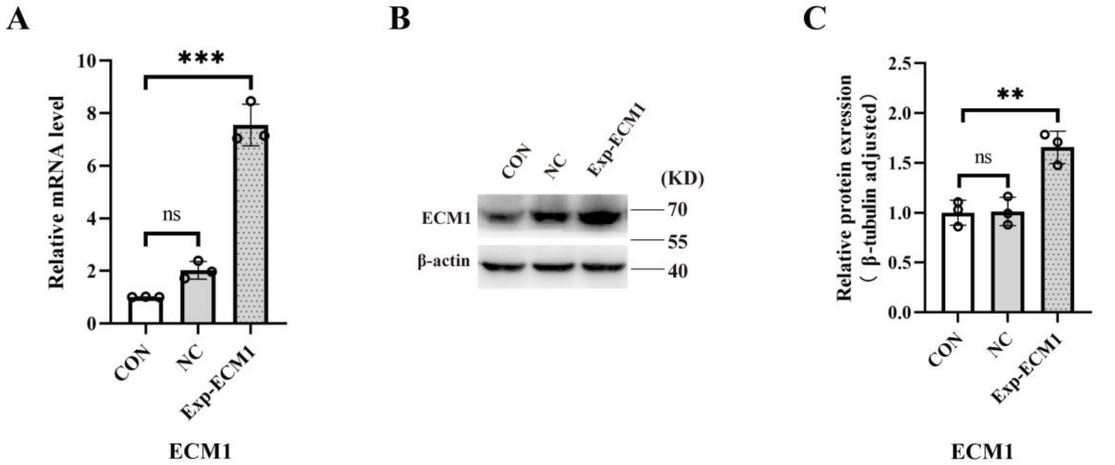

Figure S4 Constructing an ECM1 overexpression Raw 264.7 cell line. (A) The mRNA expression of ECM1 in each group of cells; (B) The protein expression level of ECM1 in each group of cells; (C) Quantitative determination of ECM1 protein expression; Statistical differences in multiple groups were determined by one-way ANOVA followed by Tukey's multiple comparisons. All data is represented as mean ± SD, n=3. **: $p < 0.01$, ***: $p < 0.001$, vs. CON. ns: $p > 0.05$.

Table S1 Primers List

Gene	primer sequences
F-iNOS	ATCTTGGAGCGAGTTGTGGATTGTC
R-iNOS	TAGGTGAGGGCTTGGCTGAGTG
F-Arg1	AGACAGCAGAGGAGGTGAAGAGTAC
R-Arg1	AAGGTAGTCAGTCCCTGGCTTATGG
F-IL6	CTTCTTGGGACTGATGCTGGTGAC
R-IL6	TCTGTTGGGAGTGGTATCCTCTGTG
F-TNF-α	CGCTCTTCTGTCTACTGAACTTCGG
R-TNF-α	GTGGTTTGTGAGTGTGAGGGTCTG
F-ECM1	GCGAGCTTCCATATCCAGAACAGG
R-ECM1	TCAGGAGACAGGTCACAGCAGAG
